# Stem‐Cell‐Based Small‐Diameter Blood Vessels with 3D Printing

**DOI:** 10.1002/smsc.202400261

**Published:** 2024-09-10

**Authors:** Yifan Wang, Xinhuan Wang, Jun Chen, Gordon Wallace, Qi Gu

**Affiliations:** ^1^ Key Laboratory of Organ Regeneration and Reconstruction State Key Laboratory of Membrane Biology Institute of Zoology Beijing 100101 P. R. China; ^2^ University of Chinese Academy of Sciences Beijing 100049 P. R. China; ^3^ Beijing Institute for Stem Cell and Regenerative Medicine Beijing 100101 P. R. China; ^4^ Intelligent Polymer Research Institute University of Wollongong Innovation Campus, Squires Way North Wollongong NSW 2500 Australia

**Keywords:** 3D bioprinting, regenerative medicines, small‐diameter blood vessels, stem cells

## Abstract

Cardiovascular disease has emerged as the leading cause of death worldwide. Since coronary arteries, carotid arteries, and other blood vessels are prone to narrowing, small‐diameter artificial blood channels offer a crucial solution for restoring blood flow. Ideal grafts must emulate the structure of natural blood vessels, possess adequate mechanical strength, ensure long‐term patency, and incorporate functional cells with minimal immunogenicity. Enhanced cell sources and engineering methods are vital for the creation of functional small‐diameter blood vessels (SDBVs). Among potential cell sources, stem cells stand out due to their ability to differentiate into multiple cell types, self‐renew, and exhibit low immunogenicity. Additionally, three‐dimensionally (3D) printed vascular stents have attracted widespread attention for their precision and controllable bioink application. The need for tissue‐engineered blood vessels is currently rising, and innovative design concepts integrating stem cells and 3D printing present promising solutions. Herein, the construction requirements of vascular grafts are reviewed, current status of using stem cells as a cell source and 3D printing as an engineering strategy is described, and prospects and challenges for the development of SDBVs in the medical field are discussed.

## Graphical abstract

Herein, the use of 3D bioprinting technology to construct stem‐cell‐based small‐diameter blood vessels is reviewed. In this review, 3D bioprinting is focused on and various stem cell sources , as well as their differentiation into endothelial cell and smooth muscle cell, are highlighted. This integration aims to meet clinical needs in vascular tissue engineering.

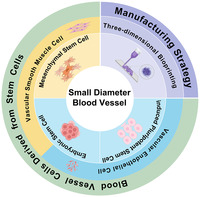

## 1 Introduction

**Figure 1:**
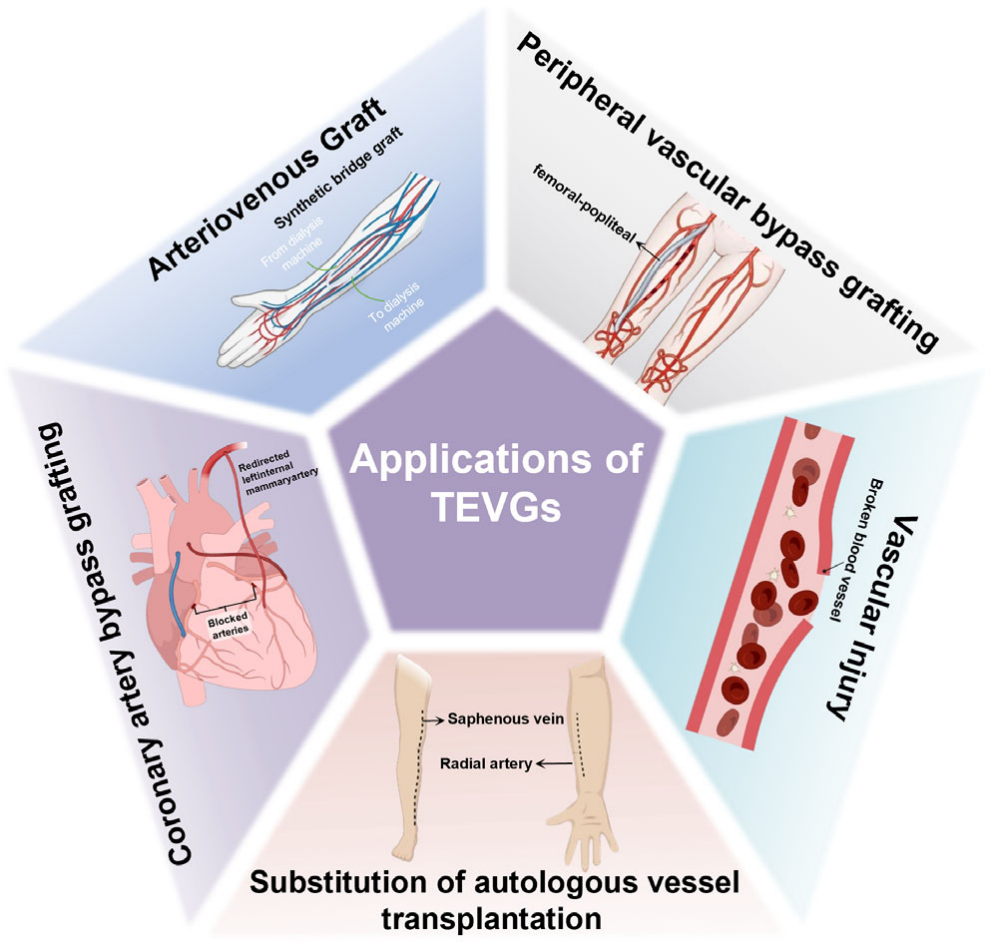
Extensive clinical needs of tissue engineering small‐diameter blood vessels.

According to a 2021 World Health Organization report,^[^ ^1^ ^]^ cardiovascular disease (CVD) is a noncommunicable disease with the highest proportion of total deaths. CVD is the leading cause of death globally, with one‐third of all deaths occurring in people under the age of 70. Among CVD subsets, atherosclerosis CVD (ASCVD) is the leading cause of death.^[^ ^2^ ^]^ The typical formation of ASCVD includes intimal hyperplasia, necrotic tissues, aberrant lipid, calcium, and macrophage deposition, which leads to arterial obstruction and blood supply loss.^[^ ^3^ ^]^ The clinical requirements of tissue‐engineered vascular grafts (TEVGs) are demonstrated in **Figure** **1**. Furthermore, as ASCVD tends to accumulate in narrow areas of coronary arteries, carotid arteries, and other blood vessels, autologous vascular transplantation is currently the main intervention method in clinical practice.^[^ ^4^, ^5^, ^6^ ^]^ ≈20–30% of patients lack autologous vessels suitable for use in transplants.^[^ ^7^ ^]^ Therefore, due to the limitations of autologous blood vessel size, secondary trauma, and limited usable area, artificial small‐diameter blood vessels (SDBVs) are still considered to be better with broad application prospects.^[^ ^8^ ^]^

**Figure 2:**
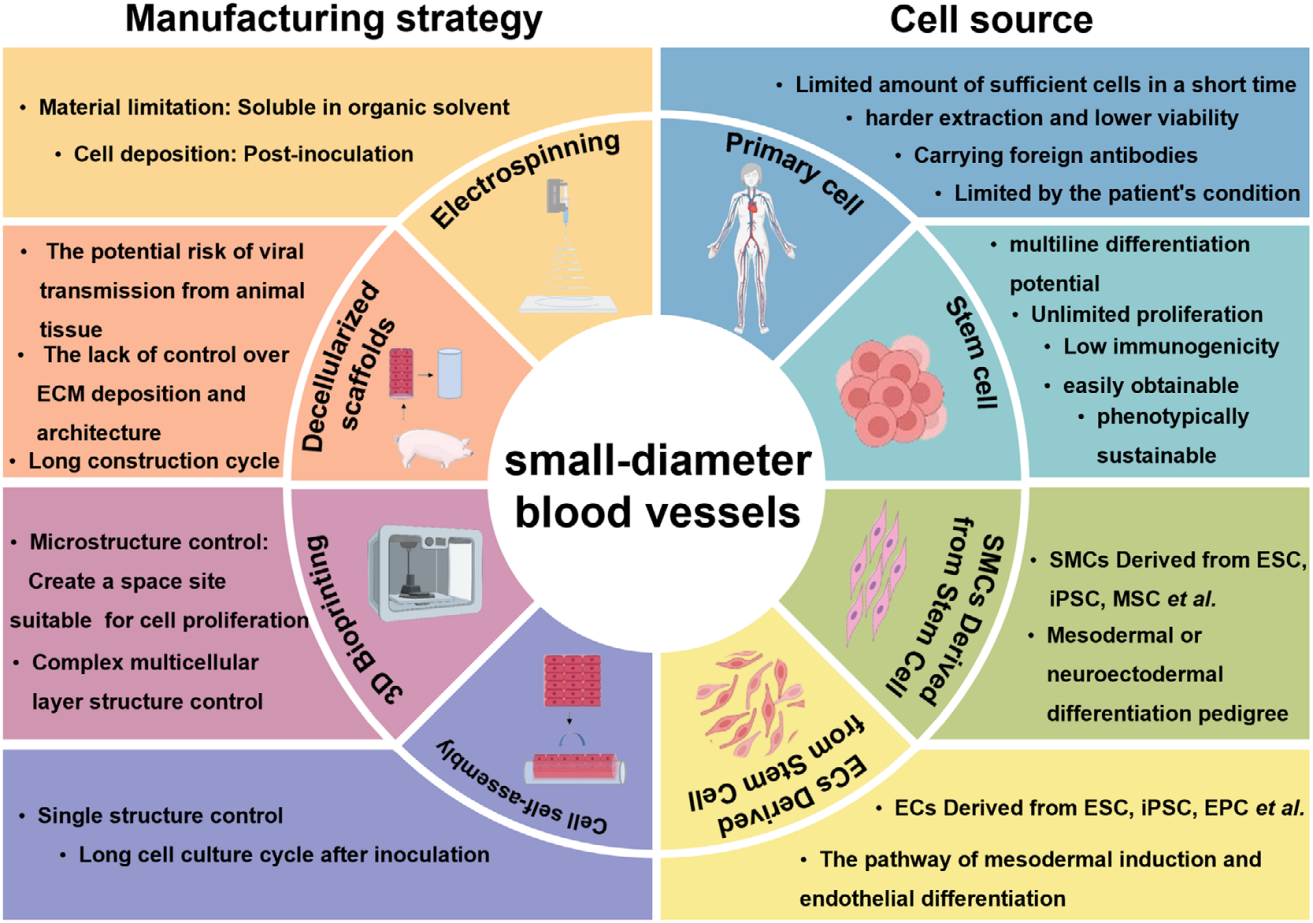
Overview of manufacturing strategy and cell source for SDBVs. Reproduced with permission.^[^ ^153^ ^]^ Copyright 2020, published by John Wiley and sons.

To create artificial SDBVs, tissue engineering methods have been widely applied to regulate the behavioral activities of seed cells using scaffold materials and to manipulate a series of growth factors to form vascular tissues with specific structures and functions in vitro (**Figure** **2**). Numerous research findings have demonstrated the benefits of unrestricted diameter length, robust wall support, and good compression effect at joints,^[^ ^9^, ^10^, ^11^, ^12^, ^13^ ^]^ and research on large‐diameter blood vessel (>6 mm) technology has evolved.^[^ ^14^ ^]^ However, there has not been any achievable breakthrough in constructing SDBVs. Hemodynamics is essential for vascular homeostasis and remodeling.^[^ ^15^ ^]^ Blood flow behavior and other mechanical characteristics are different in SDBVs compared to that in large‐diameter vessels.^[^ ^16^ ^]^ Blood flow reduction facilitates inducing wall hypertrophy or hyperplasia of the intimal intermediate, which further narrows the blood vessel radius and causes imbalanced vascular homeostasis.^[^ ^17^ ^]^ An intact, functional endothelium and its capacity to undergo adaptive changes in structure and function in response to shear stress modifications are essential for the compensatory arterial response to changes during arterial remodeling.^[^ ^18^ ^]^ As a result, clinical uses for SDBVs are limited.^[^ ^1^ ^]^

Tissue engineering vascular scaffolds are commonly constructed using decellularization,^[^ ^10^, ^19^ ^]^ 3D bioprinting,^[^ ^20^ ^]^ electrospinning,^[^ ^10^ ^]^ and self‐assembly molding.^[^ ^21^ ^]^ A comparison of commonly used types of tissue engineering is shown in **Table** **1**. Acellular vascular scaffold refers to the vascular scaffold composed of extracellular matrix (ECM) after the decellularization of natural blood vessels or allogeneic and xenogeneic cells deposited by the ECM, which is then decellularized to obtain a vascular scaffold composed of ECM. The overall objective of decellularization is a trade‐off between achieving a high degree of decellularization and preserving the structure and properties of the ECM. Furthermore, the optimal degree of decellularization depends on the properties of the native tissues and the intended use of the matrix.^[^ ^22^ ^]^ However, decellularization treatment is not only complex and time‐consuming, but also reduces the mechanical strength of autologous blood vessels.^[^ ^9^, ^23^, ^24^ ^]^ Notably, although the antigenicity of the acellular scaffold is removed to a certain extent, the ECM endotoxin still limits the safety of a wide range of allogeneic or xenograft transplants.^[^ ^25^ ^]^ In addition to acellular scaffolds, another kind of scaffold in the field of SDBVs is defined as polymer vascular scaffold, which is manufactured as the aforementioned 3D printing, etc. As general technologies such as electrospinning have been reported in detail in previous reviews,^[^ ^22^, ^26^, ^27^, ^28^, ^29^, ^30^, ^31^ ^]^ this review focuses on the application of 3D‐printing technology in SDBV manufacturing.

**Table 1:** Comparison of common tissue engineering methods for SDBVs.

Comparative item	3D bioprinting	Electrospinning	Cell self‐assembly	Decellularized scaffolds	References
Material selection	Printability	Solubility of organic solvent	ECM	Allogeneic vessel	[141, 142, 143, 144]
Structure control	Complex	Normal	Simple	Simple	[9, 124, 145, 146, 147]
Construction duration	Short	Short	Long	Long	[10, 143, 144, 148, 149, 150]
Cell deposition	Embedded	Post‐inoculation	Post‐inoculation	In vivo	[21, 115, 146, 151]

Compared with acellular vascular stents, 3D‐bioprinted vascular stents have attracted extensive attention due to their precise and controllable bioink performance. The 3D printing is the process of positioning and assembling biological materials or cell units, according to the principle of additive manufacturing and manufacturing tissue‐engineered organs and other products driven by digital 3D models.^[^ ^32^ ^]^ Currently, 3D bioprinting technology for tissue‐engineered blood vessels is mainly divided into hydrogel vascular stents and polymer vascular stents based on their bioink components. Hydrogel vascular scaffolds use biomaterials such as hydrogels loaded with live cells as temporary support for early cell growth to induce cell localization, maturation, and differentiation, but long‐term stability requires mechanical strength that matches the autologous vascular anastomosis.^[^ ^33^ ^]^ As a result, hydrogel materials are limited in their application to SDBVs. Polymer vascular stents could better manage material qualities by adjusting mechanical properties, porosity, and degradation rate based on actual needs, thereby regulating cell adhesion, migration, proliferation, and differentiation. Unfortunately, polymer materials typically do not have the same biological activity as naturally occurring biological components, which prevents them from offering seed cells a favorable environment for adhesion and proliferation. This ultimately results in poor vascular remodeling.^[^ ^34^ ^]^ Therefore, the loss of functional cells has been identified as a major factor in the failure of 3D printing to fabricate tissue‐engineered blood vessels.^[^ ^35^ ^]^

The success of tissue‐engineered blood vessels is primarily dependent on the capacity of cells to reconstruct their original tissue composition and structure.^[^ ^36^ ^]^ Vascular endothelial cells (ECs) play a substantial role in SDBVs by performing biological functions and maintaining vascular patency. The ECs act as physical barriers, inhibit the abnormal migration of smooth muscle (SM) cells, and regulate blood coagulation.^[^ ^36^ ^]^ Endothelialized tissue‐engineered grafts have been proven to maintain patency for longer periods of time in vivo. A lack of an endothelium layer causes protein deposition, platelet activation, and complement response on the surface of the biomaterial.^[^ ^37^ ^]^ Therefore, it is especially critical that ECs spread logically throughout tissue‐engineered blood vessels. The main cell type in tissue‐engineered blood vessels is vascular SM cells (VSMCs), which is responsible for contractility and ECM deposition, both of which are critical for preserving the mechanical properties of vascular grafts. Therefore, developing a tissue‐engineered blood vessel that can adjust to the fluid flow in the tube and persist steadily for a long period through the type of seed cells that fit within is challenging.

Seed cells are usually primary cells isolated from the patient's vascular tissue in early vascular tissue engineering. However, primary cells face issues, including harder extraction and lower viability, when the number of expansion generations increases. Furthermore, the availability and viability of these cells are limited by the donor's vascular conditions, and elderly patients with systemic CVD would face serious cell source problems.^[^ ^38^ ^]^ However, the same or xenogeneic primary cells trigger the body's immune response due to the carrying of foreign antigens, and are unable to live steadily in the patient's body for an extended period of time. In contrast to the vascular SM layer, which can be decellularized after ECM deposition, ECs must remain intact in functional tissue‐engineered vessels. Therefore, the cell types used in vascular tissue engineering must be immuno‐compatible. Stem cells are capable of generating any cell types owing to their low immunogenicity, the ability to self‐renew, and capacity for pluripotent differentiation. Additionally, stem cells also provide sufficient and timely cell sources for vascular interventional surgery in an emergency. Therefore, the application of stem cells as seed cells in vascular tissue engineering is increasingly becoming the focus of research. Embryonic stem cells (ESCs), human‐induced pluripotent stem cells (hiPSCs), mesenchymal stem cells (MSCs), and other sources could be used for vascular tissue engineering.

**Figure 3:**
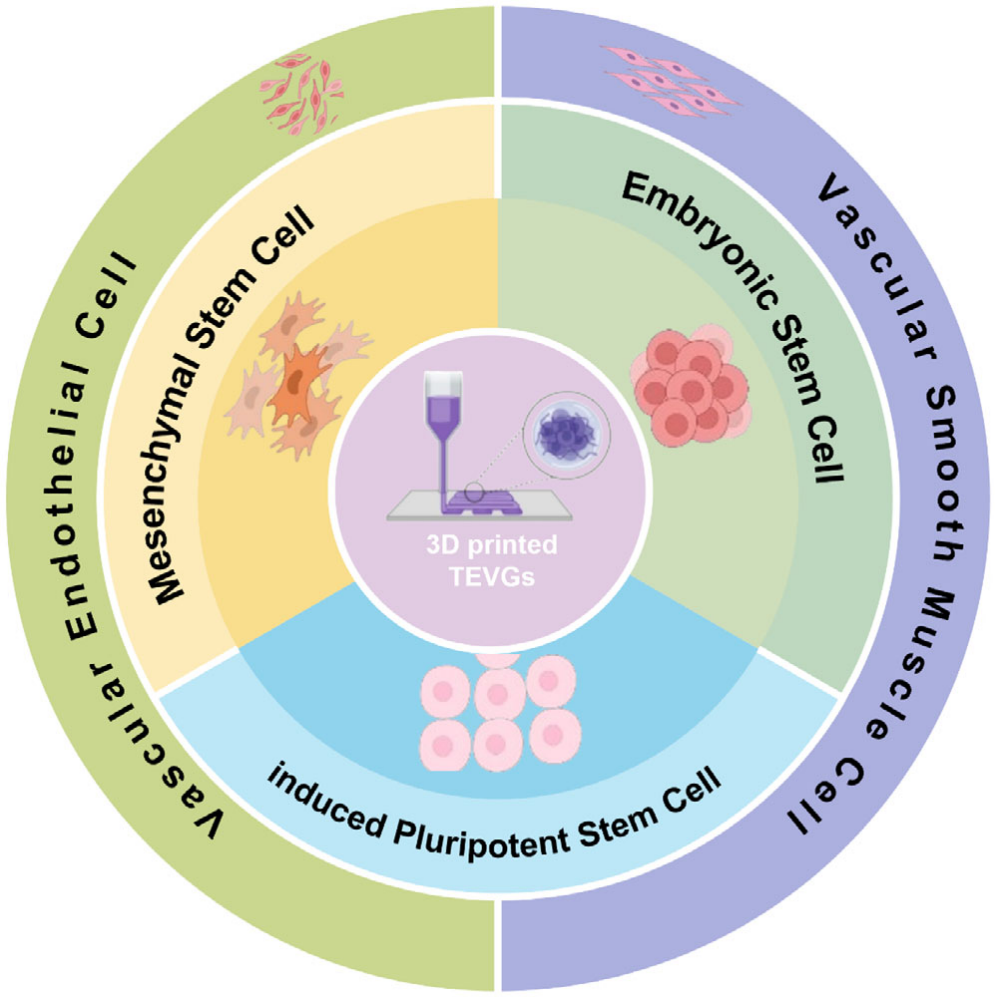
Diagram of 3D‐printed vascular tissue technology and cell source.

With the advancement of vascular tissue engineering, specifically stem cells, a safe and efficient supply for autologous vascular cells and 3D‐printing technology, capable of constructing multilayer tissues, may work collectively to solve the challenging issues of SDBV construction (**Figure** **3**). In this review, we describe strategies for 3D‐printing SDBVs based on stem cells. We also examine recent advances in the use of stem cells as seed cells and discuss new developments for fabricating small‐diameter vascular grafts.

## 2 Basic Requirements of SDBVs

### 2.1 Key Structures and Cellular Components

Vascular tissue engineering aims to design living, responsive vessels with characteristics close to those of the natural tissues. SDBVs must functionally meet the physiological requirements of human vasculature.^[^ ^27^ ^]^ To manufacture blood vessels in vitro, replication of the three‐layer structure of the natural blood vessel wall, ensuring the structural integrity of each layer and defining the ordered combination of functional division of labor, is required, to maintain the long‐term stability of the blood vessel wall. Three main cell types make up the vascular wall (apart from capillaries), which has a complex architecture and distinct mechanical properties: ECs lining the tunica intima, SMCs found in the tunica media, and adventitial fibroblasts in the tunica adventitia. Among these, ECs are essential to preserving the vessel's mechanical properties and integrity. For instance, ECs regulate the migration of parietal cells by sensing stress and stimulating physical force. Platelet‐derived growth factor–BB (PDGF–BB), which is secreted by paracrine pathways like the PDGF‐signaling pathway, ensures the long‐term stability and function of blood vessels. Additionally, ECs promote organ repair and regeneration by maintaining the homeostasis of particular tissue stem cells.^[^ ^39^ ^]^ Furthermore, the endothelium layer offers a thrombo‐resistant, continuously selectively permeable barrier that facilitates laminar blood flow via the blood vessel. The endothelium layer also controls vascular tone, platelet activation, adhesion and aggregation, leukocyte adhesion, and SMC migration and proliferation. Meanwhile, SMCs possess secretory abilities. The elasticity and radial compliance of the vasculature are maintained by the collagen fibers, elastic fibers, elastic lamellae, and proteoglycans secreted by the SMCs.^[^ ^40^, ^41^, ^42^ ^]^

**Figure 4:**
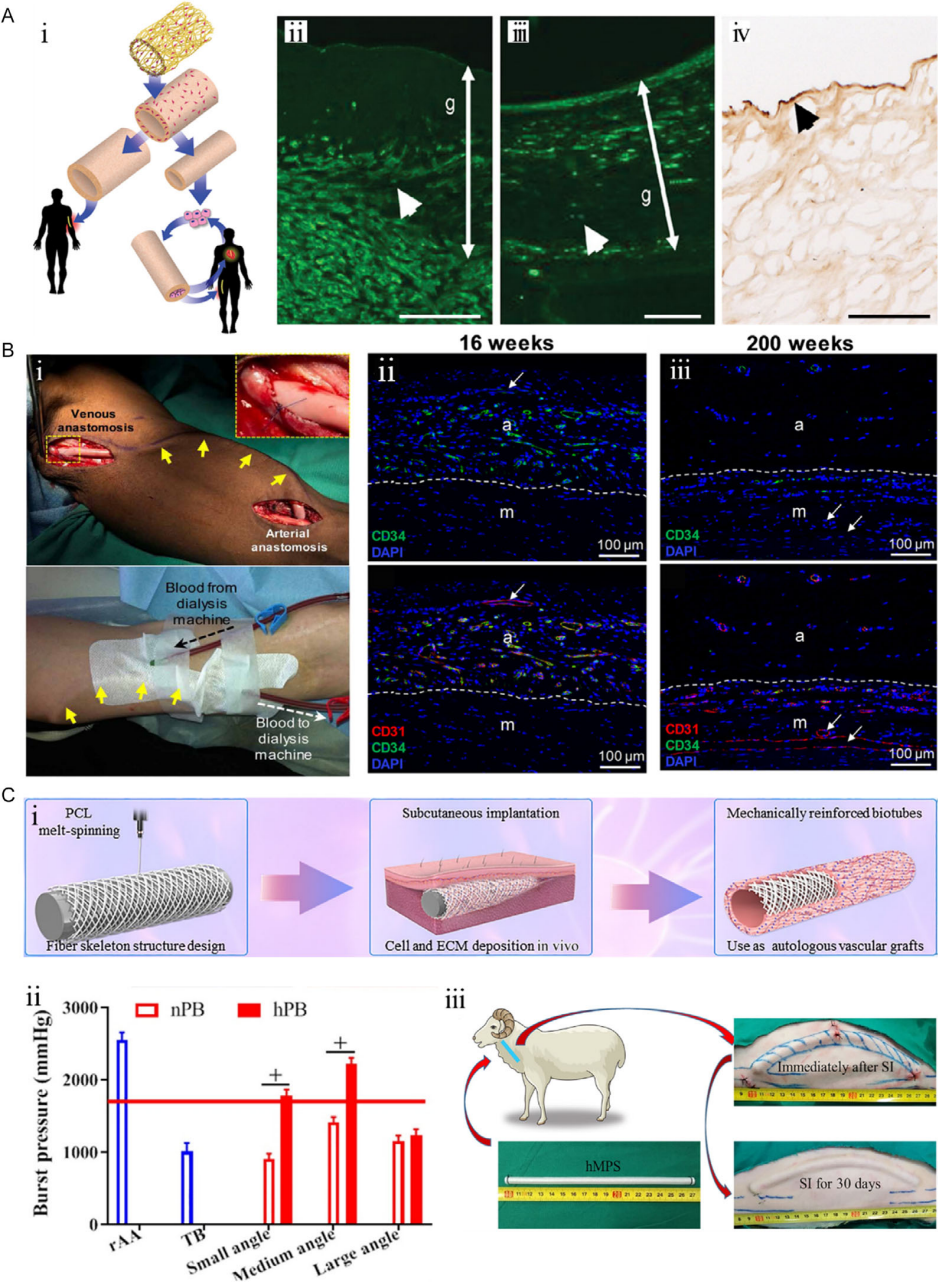
The strategy of constructing SDBV meeting the basic requirements of vascular transplantation. A) i) SDBV manufacturing strategy with complete key structure and cell composition. ii) Immunofluorescence images of vascular wall structure composed of SMCs 1 month after transplantation in dog arterial bypass grafting model. iii) At a year, complete SMCs‐like structures similar to natural blood vessels exist in the blood vessel wall. iv) Hematoxylin‐eosin staining of vWF positive cells in baboon model at 6 months after transplantation. Scale bars, 100 μm. Reproduced with permission.^[^ ^10^ ^]^ Copyright 2011, American Association for the Advancement of Science. B) i) SDBVs were used for hemodialysis puncture in patients with end‐stage renal failure from 4 to 8 weeks after implantation. ii,iii) The αSMA‐positive cell population gradually migrated to the inside of SDBV and the expression of CNN1, a marker of mature contractile SMC, was upregulated. Nuclei were counterstained with 4',6‐diamidino‐2‐phenylindole (DAPI). Scale bar, 100 μm. Reproduced with permission.^[^ ^11^ ^]^ Copyright 2019, American Association for the Advancement of Science. C) i) Schematic diagram of mechanical enhanced biotube strategy imitating construction engineering. Quantitation of and burst pressure of rAA, TB, nPB, and hPB (*n* = 5). The red line denotes 1600 mmHg. Statistical significance was calculated by two‐way analysis of variance with Tukey's test. The symbol “#” denotes the comparison between different groups. The symbol “+” indicates the comparison in the same group. ii) #*p* < 0.05 and ###*p* < 0.001. iii) Performance evaluation of AVG in sheep model. Reproduced with permission.^[^ ^9^ ^]^ Copyright 2022, American Association for the Advancement of Science.

The acellular blood vessels prepared by Niklason et al. facilitated the formation of structures similar to natural blood vessels in the canine model^[^ ^10^ ^]^ (**Figure** **4**A). After 1 year of remodeling in vivo, a medial layer of α‐SM actin (α‐SMA)‐positive cells and a layer of von Willebrand factor (vWF)‐positive ECs were gradually formed. Acellular human SDBVs as a hemodialysis pathway in patients with end‐stage renal disease can achieve different degrees of multicellular vascular wall remodeling in the range of 16–200 weeks^[^ ^11^ ^]^ (Figure 4B). Therefore, SDBVs with reproducible vascular cell components and a multilayer complex structure have important research significance as clinical grafts.

### 2.2 Mechanical Properties

The primary functional of this construct is to support blood flow; therefore, it must have the proper mechanical properties to bear variations in hemodynamics in vivo. The construct must withstand hemodynamic stress over an extended period of time without failing and not be susceptible to enduring creep that could result in aneurysm.^[^ ^43^ ^]^ The mechanical requirements of small‐diameter SDBVs include long‐term fatigue resistance, biomimetic vessel compliance, sufficient burst pressure, high anastomotic suture retention strength, and mechanical homogeneity. Grafts have been shown to resist fatigue without noticeable dilatation during xenogeneic implantation and to cyclic physiological loads for 30 days in vitro.^[^ ^22^ ^]^ In addition, grafts should possess suitable compliance to prevent the formation of high stresses around the anastomosis.^[^ ^44^ ^]^ To match the cyclic nature of pulsatile flow in vivo, compliance should be between 0.7 and 1.5.^[^ ^45^ ^]^ Wall collapse can be avoided catastrophically with sufficient burst pressure. To mimic native tissue, engineered vascular grafts should have a burst pressure ≥1700 mmHg, which is equivalent to that of the saphenous vein.^[^ ^46^ ^]^ The suture retention strength of native vasculature varies based on vessel type and location, evaluated by the force required to dislodge a suture from the surgical anastomotic region.^[^ ^27^ ^]^ The human saphenous vein can retain strengths in the range of 196 ± 2 gf.

Inspired by construction engineering, Kong's team designed PS‐reinforced biotubes (PBs) that combined polycaprolactone (PCL) fiber skeletons (PSs) spun by melt with tissue engineering to compensate for the lack of mechanical properties of traditional biotubes (TBs)^[^ ^9^ ^]^ (Figure 4C). In the construction strategy, heat treatment and differing fiber winding angles are the key link for controlling the mechanical strength of PBs. Compared to non‐heat‐treated PBs (nPBs), heat‐treated PBs (hPBs) show mechanically enhanced fibrous fusion. Their study revealed that heat treatment made PBs exhibit a stronger fusion trend at the fiber crossover points, which provided adequate mechanical support. In instances where pore size is positively correlated with filament winding angle, medium‐angle hPBs (hMPBs) demonstrated a comprehensive advantage in suture retention and burst pressure, even equal to or better than that of the rat abdominal artery (rAA). The mechanical properties of hMPBs were still by‐and‐large maintained after several puncture tests in the sheep arteriovenous graft (AVG) model, which is more prominent than the sheep carotid artery. Furthermore, the neointima and calponin (contractile SMC marker) tissue structure in hMPBs three months after AVG (hMPB‐A3m), which was endowed with sufficient mechanical strength to resist blood flow pressure, indicated impressive vascular remodeling.

### 2.3 Endothelization for Long‐Term Durability

The most major clinical constraint to the long‐term durability of vascular grafts is the progression of graft occlusion overtime.^[^ ^44^ ^]^ One of the primary causes of artificial graft failure is an incomplete endothelium.^[^ ^35^ ^]^ Numerous studies have shown that SDBVs can be successfully implanted, and that EC migration from native tissue can endothelialize the lumen. However, many studies have shown prolonged times of cellular infiltration, recruitment, adhesion, remodeling, and resorption of graft materials, which can impede the system's complete integration. The Niklason's group reported that their acellular engineered arteries appear to rebuild with recipient cells overtime.^[^ ^10^ ^]^ However, after a 16‐week remodeling process, partial biopsies of the engineered blood vessels showed that the host cells reappeared with SM cells or myofibroblasts and ECs on the lumen. Vascular embolism or bleeding risk is markedly elevated in the event of an extended host cell remodeling period. Thus, to ensure long‐term patency in vivo, vascular grafts require an intact endodermis that can rebuild quickly.

### 2.4 Biocompatibility of Graft Materials

The properties of SDBVs should be suitable for implantation.^[^ ^44^ ^]^ Severe immunological rejection in vivo due to low immunogenicity should be avoided to reduce inflammation and avoid rejection. This is reflected in the immuno‐compatibility of materials and cells. Both natural biomaterials and synthetic materials are widely used as bioinks in 3D‐printed vascular grafts.^[^ ^47^, ^48^, ^49^, ^50^, ^51^ ^]^ The former was generally more bioactive than synthetic polymers, with many of them exhibiting favorable qualities for vascular applications.^[^ ^52^ ^]^ These include conventional ECM components,^[^ ^41^ ^]^ such as collagen and elastin, where recent advances offer renewed promise, as well as non‐mammalian macromolecules like silk, cellulose, and chitosan. However, issues often associated with these sources, such as lack of mechanical strength and rapid degradation, have limited the future development of organically derived graft replacements.^[^ ^53^ ^]^ Polyglycolic acid (PGA), polylactic acid, and PCL are three of the most widely utilized synthetic polymers.^[^ ^54^ ^]^ The rough and hydrophobic surface of synthetic materials that usually with faint EC layer typically results in enhanced blood cell interactions and protein adsorption, which accelerates the formation of clots.^[^ ^51^ ^]^ More importantly, ensuring that the degradation products of biodegradable synthetic materials are biocompatible and do not cause irreversible effects on the osmotic pressure of the surrounding tissue is required.

## 3 Vascular Cells Derived from Stem Cells

Vascular tissue engineering involves an intricately orchestrated series of cellular and molecular events that engage vascular progenitor cells and nonvascular cells in a specialized microenvironment. Cross talk between ECs and pericytes or VSMCs is essential for blood vessel function.^[^ ^55^ ^]^ Perivascular cells such as pericytes and SMCs are essential for stabilizing and maturing blood vessels. Cells that are non‐immunogenic, phenotypically sustainable, and easily obtainable are required for tissue‐engineered blood vessels to be used in clinical surgery. The safest source of cells for patients is autologous tissue. To avoid the restricted supply of autologous cells, Weinberg and Bell first tried to fabricate a biological vascular graft with xenogeneic cells embedded in 1986.^[^ ^56^ ^]^ They cultivated bovine fibroblasts, VSMCs, and ECs on a collagen gel substrate to replicate the adventitia, media, and intima layers of the vessel, respectively. However, the limited accessibility, finite expandability, donor–donor functional variation, and immune incompatibility of primary seed cells from donors may greatly impede the application of SDBVs.^[^ ^57^ ^]^

With great success, the source of cells has changed to stem cells as an alternative.^[^ ^55^ ^]^ These cells appear to be minimally immunogenic, expressing only moderate levels of major histocompatibility complex (MHC) class I and not any MHC class II proteins.^[^ ^58^ ^]^ Furthermore, human stem and progenitor cells have been isolated from a wide range of sources. Their autologous origin, high proliferative capacity, and ability to differentiate into vascular phenotypes have generated considerable interest.^[^ ^59^ ^]^ Overcoming cell access limitations relies on the multi‐lineage differentiation and self‐renewal abilities of stem cells. Human stem cells for disease modeling in vitro and clinical applications require protocols converting them into relevant adult cell types.^[^ ^60^ ^]^

### 3.1 ECs Derived from Stem Cells

#### 3.1.1 ECs Derived from Induced Pluripotent Stem Cells

Vascular cells can be obtained from induced pluripotent stem cells (iPSCs) derived from heathy individuals and patients and these cells do reflect the genetic background of the original person.^[^ ^61^ ^]^ Under external stimuli, iPSCs are capable of differentiate into any of the three germ layers’ cells. iPSC‐derived vascular cells display similar features to mature vascular cells at the genetic and functional levels.^[^ ^62^ ^]^ Yamanaka's group was the first to find that iPSC cells could be obtained by inducing four pluripotency factors (OCT‐3/4, Sox2, Klf4, and c‐Myc); these cells could be utilized to reprogram somatic cells that can differentiate into any cell type in the three germ layers under specific conditions.^[^ ^63^ ^]^

ECs can be differentiated from iPSCs through three methods: stromal cell coculture, embryoid body (EB) differentiation, and feeder‐free monolayer differentiation.^[^ ^64^ ^]^ The stromal cell coculture method could not separate ECs, stromal cells, and other differentiated cell types (such as SMCs), and it exhibits a low degree of cell differentiation, which is overly dependent on the quality of stromal cell lines and serum.^[^ ^65^ ^]^ The most common method used for differentiation relies on the generation of EBs through the spontaneous differentiation of aggregated hiPSCs within the framework of a self‐assembled 3D structure.^[^ ^66^ ^]^ As the differentiation of EBs is not a fully controlled process and ultimately results in lower EC production, less than 50% of ECs can be derived from iPSCs.^[^ ^62^, ^67^ ^]^ As a result, the feeder‐free monolayer differentiation method has gradually entered the field. Feeder‐free monolayer differentiation is an iPSC differentiation strategy that combines different culture substrates and chemical conditions.

Orlova's group provided chemical signal stimulation using substances such as bone morphogenetic protein or vascular EC growth factor to complete differentiation in the stages of mesoderm induction and endothelial differentiation, respectively.^[^ ^64^ ^]^ iPSC‐derived ECs showed typical endothelial morphology with the expression of endothelial‐specific markers of VE‐cadherin, platelet endothelial cell adhesion molecule‐1 (PECAM‐1/CD31), and vWF. In zebrafish xenotransplantation experiments, ECs derived from iPSCs are capable of vascularization in vivo, which is an important embodiment of EC function. However, this approach failed to attain the target percentage of ECs in the differentiated cell population and could not demonstrate stability in multiple iPSC lines.^[^ ^68^ ^]^

**Figure 5:**
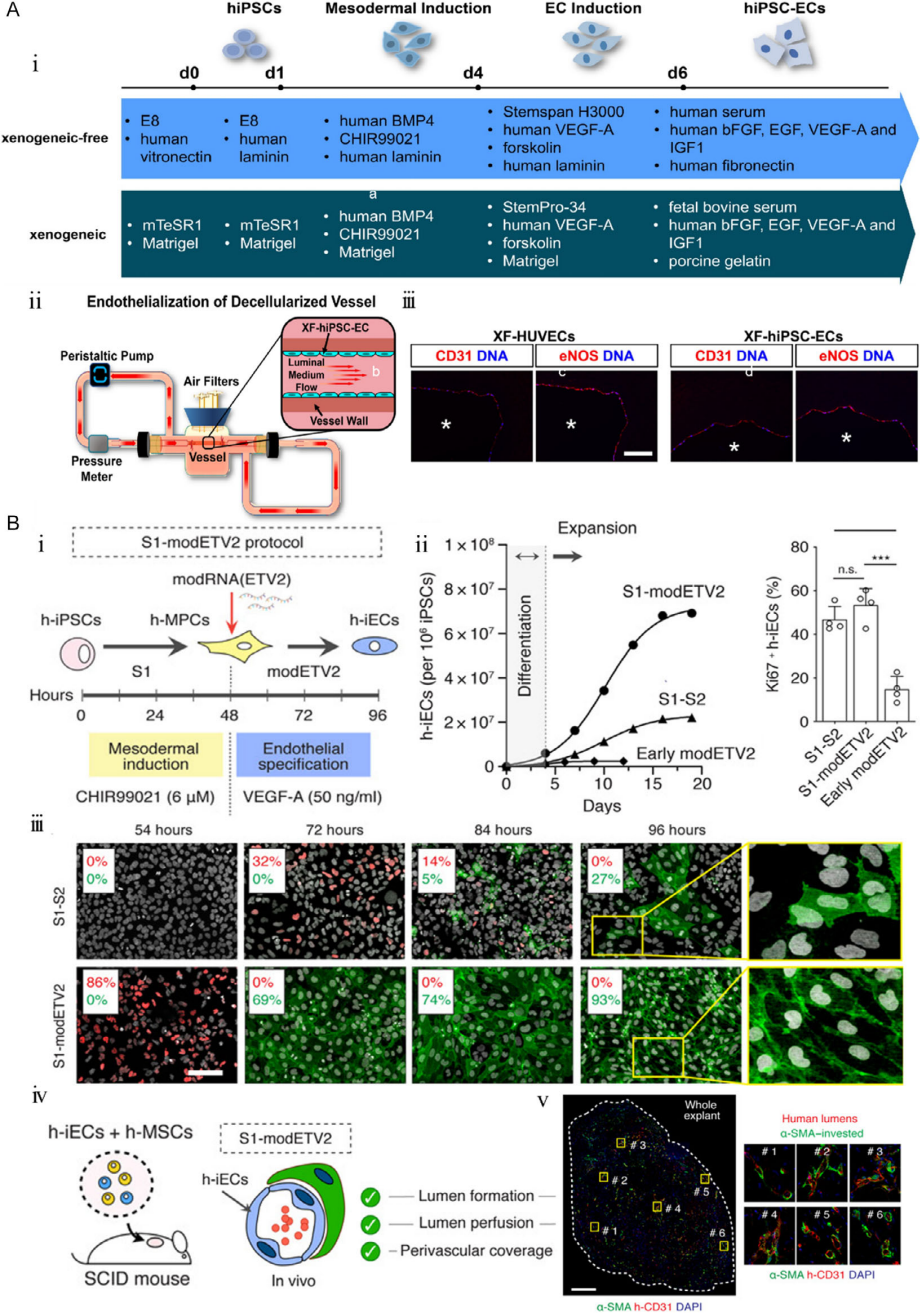
The strategy of deriving ECs from iPSC. A) i) Schematic diagram of strategy of ECs derived from hiPSC without heterogeneous genes. ii) Schematic diagram of endothelialization of human acellular vessels by XF–hiPSC–EC in dynamic bioreactor. Immunofluorescence imaging of EC markers (CD31 and endothelial nitric oxide synthase (eNOS)) in acellular vessels after 5 days of endothelialization using XF–hiPSC–EC or XF–HUVEC. iii) Asterisk denotes the lumen. Nuclear was counterstained by DAPI. Scale bar, 100 μm. Reproduced with permission.^[^ ^69^ ^]^ Copyright 2021, Elsevier. B) Schematic diagram of iEC differentiation. i) ModRNA (ETV2) was introduced at the 48th hour. ii) Amplification curve of iEC and expression statistics of Ki67. iii) Immunofluorescence staining of ETV2 and CD31 in S1‐S2 and S1‐modETV2 protocols. Scale bar, 100 μm. iv) Schematic diagram of h‐iECs transplantation and vascular network formation in SCID mouse. v) Immunofluorescence image of iEC graft 30 days after transplantation. Nuclei were counterstained with DAPI. Scale bar, 500 μm. Reproduced with permission.^[^ ^70^ ^]^ Copyright 2020, The American Association for the Advancement of Science.

Luo's group developed methods for generating functional human induced pluripotent stem cell derived‐endotheliocyte (hiPSC‐ECs) suited for vascular tissue engineering in 2012 (**Figure** **5**A). They successfully endothelialized decellularized human vessels with xenogeneic‐free hiPSC–ECs (XF–hiPSC–ECs) in a dynamic bioreactor system.^[^ ^69^ ^]^ These XF–hiPSC–ECs displayed expression of typical endothelial markers, angiogenic potential, and uptake of acetylated low density lipoprotein (ac‐LDL). They utilized XF–hiPSC–ECs to endothelialize the luminal surface of decellularized vessels. Compared to hiPSC–ECs derived from standard xenogeneic methods (XG–hiPSC–ECs), the endothelialization efficiency of XF–hiPSC–ECs was up to 64%. After 5 days of endothelialization, the endothelial structure of acellular vessels is equivalent to that of autologous vessels with XF–human umbilical vein ECs (HUVECs). Recent findings suggest that using iPSC–ECs in vascular tissue engineering is a powerful potential solution.

Martin's group focuses on solving the problem of low efficiency of iPSC‐derived EC from the level of genetic modification.^[^ ^70^ ^]^ Transcription factor ets variant transcription factor 2 (ETV2)‐modified mRNA (modETV2) is delivered during mesodermal induction (Figure 5B). Compared with the standard scheme (S1–S2 protocol), the two‐step scheme (S1‐modETV2) achieved 93% induced ECs (iEC) differentiation efficiency within 96 h of transfection, which was generally applicable in a variety of iPSC clones. And stable amplification was achieved within 3 weeks, which average amplification rate was ≈70%. After implantation in severe combined immune deficiency mice for 30 days, an extensive human vascular network was formed in vivo. Necessarily, non‐integrative transient transfection and short‐term presence (<18 h) of modETV2 expand the potential of the clinical level.

#### 3.1.2 ECs Derived from ESC

ESC is considered as a prominent cell source for safely and effectively obtaining a large number of ECs that can be used for various tissue repair and cell replacement therapies.^[^ ^61^, ^71^ ^]^ Differentiation of ESCs into the endothelial lineage is a repetition of endothelial development during the natural development of an individual. Although there are two main methods for inducing endothelial differentiation of ESCs in vitro, low efficiency of EC production often occurs due to the uncontrolled spontaneous differentiation in EBs, which is similar to what occurs in the iPSC–EC differentiation protocol.^[^ ^72^, ^73^ ^]^ Some researchers have used OP9 feeder layers and mouse embryonic fibroblasts to stimulate ESC differentiation to vascular cells.^[^ ^74^ ^]^

**Figure 6:**
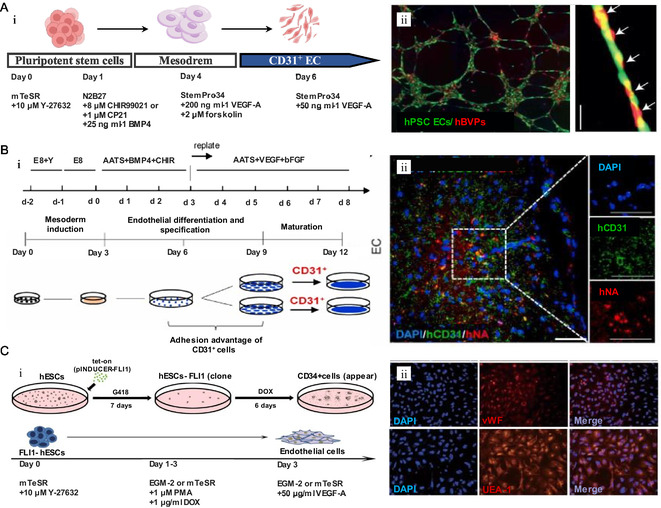
The strategy of deriving ECs from ESC. A) i) Schematic diagram of differentiation strategy of ECs derived from ESC. ii) The tubular structure of ESC–ECs and hBVPs (red) was formed on Matrigel. Scale bars, 50 μm. Reproduced with permission.^[^ ^60^ ^]^ Copyright 2015, Springer Nature. B) i) Schematic diagram of strategy for selective enrichment of EC through resurfacing step in AATS medium. ii) Immunofluorescence images of hCD31 (green) and hNA (red) in mice with middle cerebral artery occlusion (MCAO) 14 days after injecting ESC‐derived ECs in MS. Scale bar, 100 μm. Reproduced with permission.^[^ ^77^ ^]^ Copyright 2021, Elsevier. C) i) Schematic diagram of the strategy of inducing EC differentiation by FLI1 overexpression system in ESC. ii) Immunofluorescence images of endothelial markers vWF and ulex europaeus agglutinin (UEA‐1) expressed in iEC in vitro. Nuclei were counterstained with DAPI. Scale bar, 100 μm. Reproduced with permission.^[^ ^78^ ^]^ Copyright 2018, Springer Nature.

Due to the phenotypic instability and limitations of differentiation efficiency, researchers are focusing more on the introduction of engineering concepts and molecular regulatory techniques. Li's group^[^ ^75^ ^]^ established a strategy to guide the endothelial differentiation of ESCs by releasing NO in a continuous and controlled manner without the addition of growth factors. Inoculating ESCs into a controlled chitosan nitric oxide (NO)‐releasing hydrogel (CS‐NO) culture system significantly upregulated the expression of Flk‐1, an early EC marker, and triggered phosphoinositol‐3 kinase/Akt signaling. Langer's group^[^ ^76^ ^]^ developed a photopolymerizable dextran‐based hydrogel with an insoluble (Arg‐Gly‐Asp) peptides sequence or soluble vascular endothelial growth factor (VEGF) for the preferential differentiation of ESCs into vascular lineages. When VEGF extended‐release factor is added to this system, the proportion of cells expressing the vascular marker, VEGF receptor kinase insert domain receptor/fetal liver kinase‐1 (KDR/Flk‐1), increased by 22 times when compared to spontaneously differentiated EBs. Cowan's group^[^ ^60^ ^]^ reported a protocol for rapid differentiation from ESCs that could be applied to different stem cell lines with higher differentiation efficiency (**Figure** **6**A). ESCs are induced to EC or SMC lineages by the action of VEGF or PDGF–BB, respectively. The transcriptomic and metabolomic signatures of ESC‐derived ECs and SMCs are highly similar to those of their primary cells. The dependence on heterogeneous cells and serum in traditional protocols has been reduced to some extent by small‐molecule and engineering methods. Simultaneously, differentiation efficiency and time have been greatly improved. *Trans*‐endothelial electrical resistance analysis and ac‐LDL uptake experiments revealed that ESC–ECs had similar levels of function in vitro as HUVECs. The highly organized structure formed by ESC‐derived ECs and human brain vascular pericytes verifies the angiogenetic potential of ESC–ECs. In addition, the generalizable differentiation pathways leave researchers with food for thought.

Using simplified and chemically defined medium that contains only the four components of recombinant human albumin, l‐ascorbic acid 2‐phosphate, human apo‐transferrin and sodium selenite (abbreviated as AATS), an insulin‐free strategy, Na's group^[^ ^77^ ^]^ investigated the effect of insulin on two stages of iPSC differentiation: mesodermal induction (stage I) and endothelial differentiation (stage II). Insulin inhibits the production of ECs by limiting the induction stage of the mesoderm, which is common in different stem cell lines. In view of the adhesion of CD31 ^+^ECs, the reinoculation step after 6 days of differentiation effectively improved the differentiation efficiency (Figure 6B). In addition, the AATS culture system combined with porous 3D micro‐scaffolds (MSs) were able to realize a large number of differentiations of mesodermal cells into ECs on the third day. After the ESC‐derived ECs were transplanted into a mouse middle cerebral artery occlusion model, many anti‐human CD31 (hCD31)‐ and anti‐human nuclei (hNA)‐positive blood vessels were detected in the infarct cortex 14 days after transplantation, indicating that the lower limb necrosis and hemiplegia caused by ischemia were repaired or reversed.

In contrast to differentiation methods based on chemical composition definitions, Hu et al. defined a method of gene overexpression for the rapid differentiation of ESCs into ECs.^[^ ^78^ ^]^ Friend leukemia virus‐induced erythroleukemia‐1 (FLI1), a gene strongly related to endothelial differentiation, was screened from 15 transcription factors responsible for hematopoietic lineages. ESCs were induced to differentiate into iECs within 3 days by establishing an FLI1 overexpression system in ESCs and activating protein kinase C (PKC) at the same time (Figure 6C). Notably, iECs showed substantial expression of the endothelial markers vWF and Ulex europaeus agglutinin 1 in vitro. However, the clinical application of cell sources involved in gene‐level modification remains to be considered.

### 3.2 SMCs Derived from Stem Cells

#### 3.2.1 SMCs Derived from MSC

**Figure 7:**
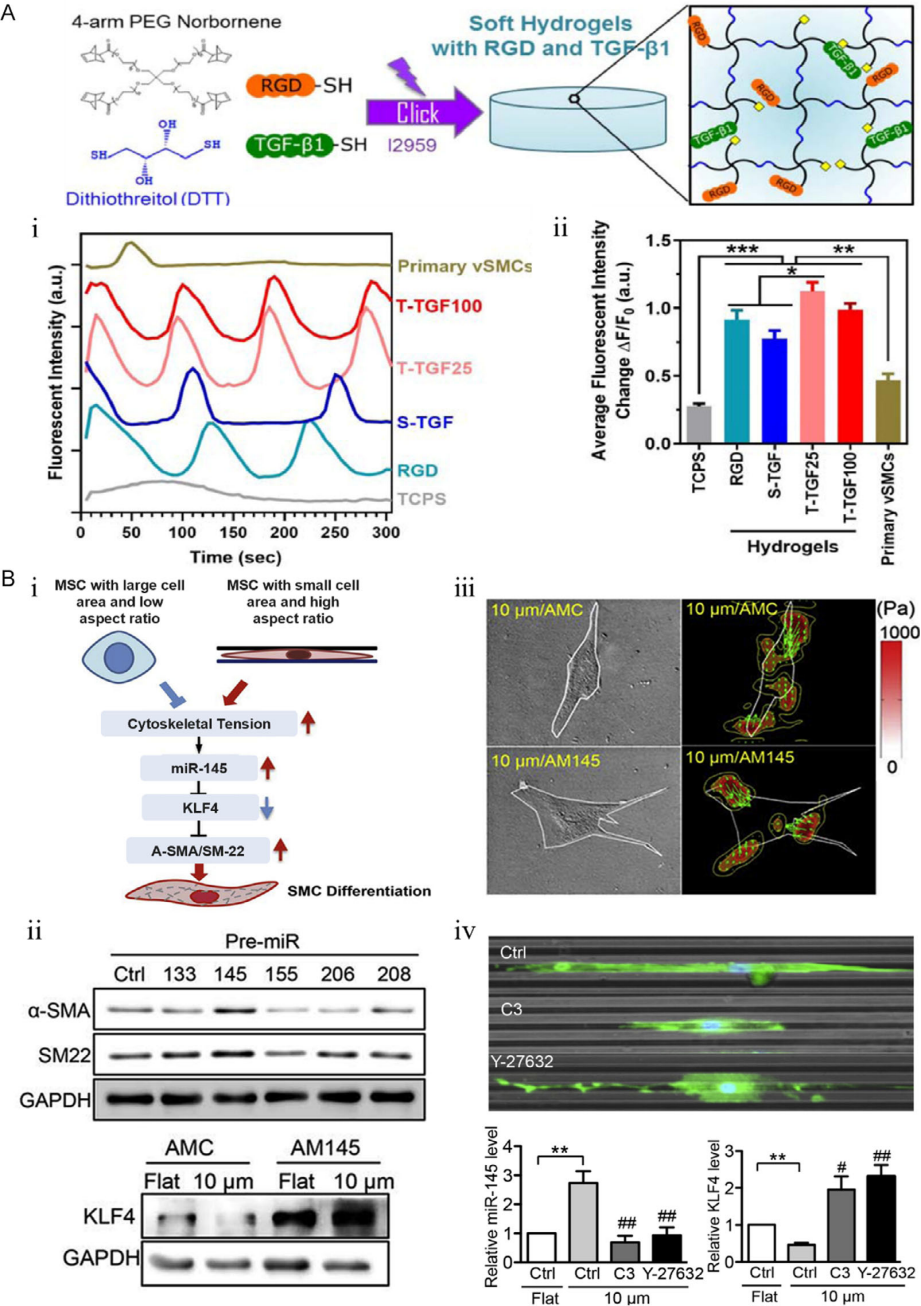
The strategy of deriving SMCs from MSC. A) Schematic diagram of hydrogel formation of tethered TGF‐β. i) Calcium oscillation image and ii) quantitative statistics. Soft hydrogel with TTGFβ1 significantly enhances the contraction function of iPSC–SMCs. Maximum fluorescent change normalized to baseline reading. *n* ≥ 40 cells per group. Reproduced with permission.^[^ ^91^ ^]^ Copyright 2020, Elsevier. B) i) Schematic diagram of miR‐145 inducing differentiation of MSC into SMC. Traction images of MSCs transfected with AM145 or corresponding controls after 3 days of culture. ii) The stress magnitude is presented by the color bar (pascal), and green arrows represent stress vector directions. Scale bar, 100 μm. iii) The expressions of α‐SMA, SM‐22, and glyceraldehyde‐3‐phosphate dehydrogenase (GAPDH) were analyzed by Western blot analysis after transfection of miR‐145 into MSCs. Representative fluorescence images of MSC morphology and statistics of miR‐145 and KLF4 expression after C3 or Y‐27 632 treatment for 24 h. Cell morphology was counterstained with fluorescein isothiocyanate (FITC)–phalloidin. iv) Nuclei were counterstained with DAPI. *n* = 3. *versus flat control, **p < 0.005. versus 10 μm microgrooved control, *p* < 0.05, *p* < 0.005. Scale bar, 20 μm. Reproduced with permission.^[^ ^93^ ^]^ Copyright 2019, Elsevier.

MSCs possess the potential for myogenic differentiation due to their capability to differentiate into different lineages of mesodermal origin.^[^ ^79^, ^80^, ^81^, ^82^, ^83^, ^84^ ^]^ Scientists have evaluated the potential of MSCs to differentiate into SMC lineages by introducing differentiation factors, mechanical force stimulation, and biomaterials. Transforming growth factor‐β1 (TGFβ1), all‐trans retinoic acid,^[^ ^85^ ^]^ PDGF–BB,^[^ ^86^, ^87^ ^]^ and basic fibroblast growth factor (bFGF)^[^ ^86^, ^87^ ^]^ are potential small molecules that promote MSC differentiation into SMCs by changing the morphology of cells.^[^ ^88^ ^]^ Studies have shown that myofibroblasts regulate TGF‐β released by MSCs during wound healing to upregulate α‐SMA expression.^[^ ^89^ ^]^ Furthermore, Moreno's group^[^ ^90^ ^]^ optimized the differentiation of MSCs into VSMCs induced by TGF‐β and BMP 4, which achieved a shortened differentiation cycle from 7 to 4 days, while maintaining the SMCs’ contraction phenotype and maintaining stable expression of SMC markers in maintenance culture for 14 days. Wei's team^[^ ^91^ ^]^ designed a polyethylene glycol hydrogel scaffold to guide MSC differentiation by providing an arterial stiffness environment and a TGF‐β teether site (T‐TGF‐X; X denotes different doses) (**Figure** **7**A). The study found that low‐stiffness hydrogels with TGFβ1 teaming exhibited higher calcium oscillations than soluble TGF‐β (S‐TGF), indicating increased calcium signaling within cells. Eventually, SM myosin heavy chain (SMMHC) became the main component of the SMC contraction‐related structure, which indicates that iPSC‐derived SMCs show greater contractility at a functional level.

miRNA, as an endogenous small molecule RNA with temporal regulation, plays an important role in the differentiation cycle of MSC.^[^ ^92^ ^]^ Chien's group designed a patterned substrate with microgrooves to induce elongation deformation of MSC, which can achieve a significant increase in the level of derived SMC markers (Figure 7B).^[^ ^93^ ^]^ Through the differentiation inhibition experiment of anti‐miR‐145 inhibitor (AM145), it was further verified that the upregulation of SMC markers was related to the upregulation of miR‐145, which enhanced the inhibition of kruppel‐like factor 4 (KLF4). The contraction characteristics of SMC were significantly inhibited after the C3 exoenzyme (a Rho inhibitor) and Y‐27 632 (a Rho‐associated coiled‐coil‐containing protein kinase (ROCK) inhibitor) treatment. The results showed that the induction of SMC contraction by miR‐145/KLF4 was inhibited by Rho/ROCK. In this study, the regulation of MSC shape by microorganisms is related to the potential molecular mechanism, which provides a broader idea for the differentiation pathway of MSC into endothelium.

In addition, physical stimuli, such as hypoxia, could induce MSCs to differentiate into SMC lineages. Studies have shown that the downregulation of Mettl3 gene expression under hypoxic conditions promotes the upregulation of the SMC markers α‐SMA, SM 22α, calmodulin, and SMMHC, as well as that of several paracrine factors, including VEGF, hepatocyte growth factor (HGF), TGF‐β, granulocyte–macrophage colony‐stimulating factor, bFGF, and stromal‐cell‐derived factor‐1.^[^ ^94^ ^]^

#### 3.2.2 SMCs Derived from iPSC

The iPSC‐derived SMC methods are comparable to the three pathways of iPSCs–ECs. Avoiding immunogenic reactions is the obvious advantage of this strategy.^[^ ^95^ ^]^ Kwong's group employed alpha‐smooth muscle actin, one of the early markers of SMCs that is broadly expressed during embryonic development, as a novel reporter gene locus to achieve a full‐time tracer during SMCs induction. The results showed that after 4 days in serum‐free medium containing bone morphogenetic protein 4, activin A, and Wnt3a, a Foxf1^+^ side plate mesoderm cell population was evident, with robust expression of the lateral plate mesoderm marker KDR. Notably, the synergistic influence of developmental signaling molecules can induce the differentiation of iPSCs into SMCs of mesodermal origin.^[^ ^96^ ^]^ This indicates the therapeutic efficacy of ESC‐derived SMCs in neovascularization and tissue regeneration.

**Figure 8:**
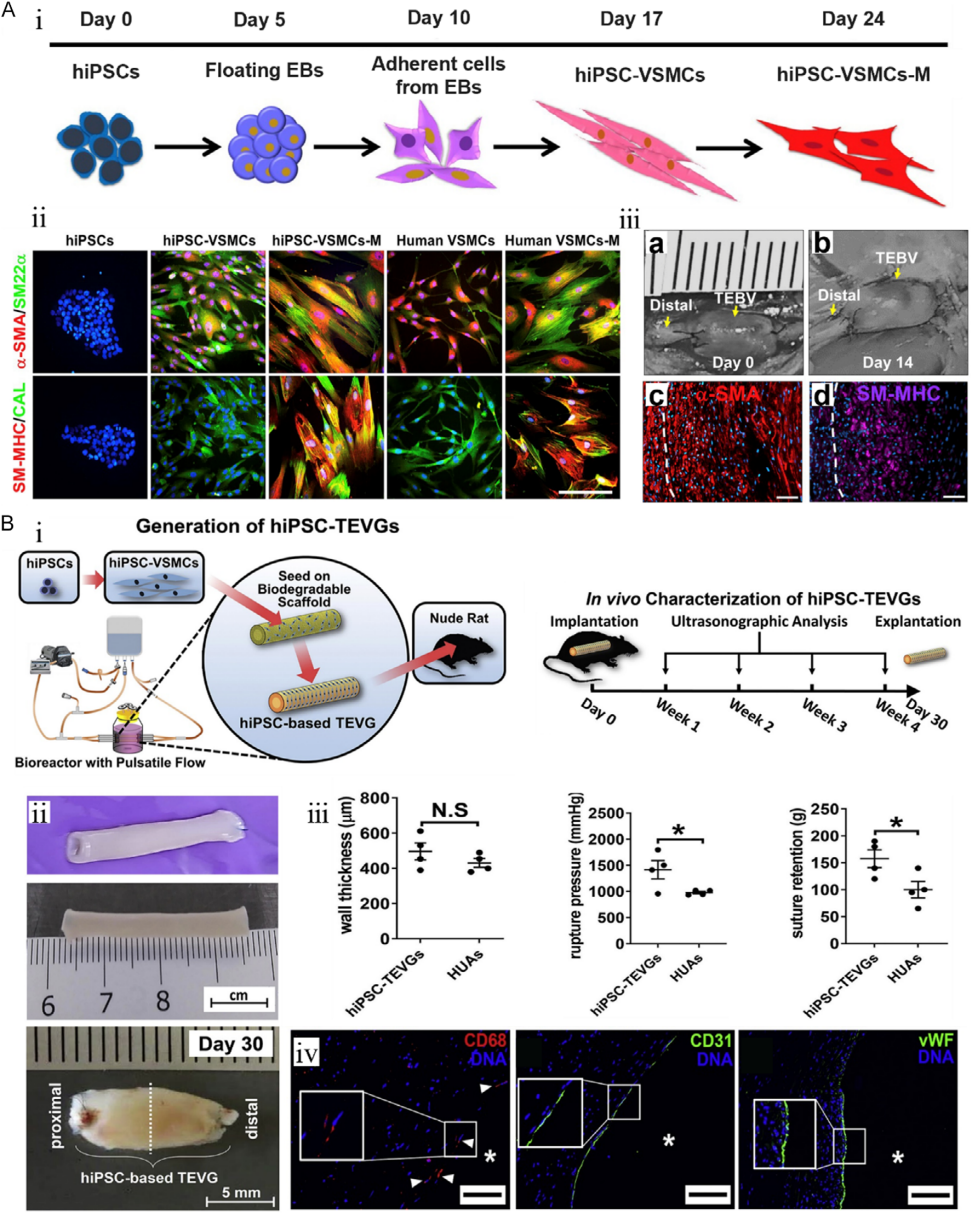
The strategy of deriving SMCs from iPSC. A) Schematic diagram of iPSC differentiation. i) Matured VSMCs (iPSC–SMC–M) were harvested by incubating iPSC–SMCs in mature medium for 7 days. ii) Immunofluorescence staining images of hiPSC–VSMC showed SMC specific markers including α‐SMA, SM22α, SM–MHC, and calponin. Scale bar, 500 μm. iii) Representative image of SDBV a) before and b) after 2 weeks of abdominal aorta transplantation in rats. Expression of SMC markers including c) α‐SMA and d) SM–MHC. Scale bar, 50 μm in (a,b). Reproduced with permission.^[^ ^97^ ^]^ Copyright 2016, Elsevier. B) i) Schematic diagram of small‐diameter blood vessel manufacturing and transplantation. ii) Representative images of small‐diameter blood vessels cultured in bioreactor with pulsatile flow for 8 weeks and after 30 days of transplantation in vivo. Maintenance of mechanical morphology of vascular wall. iii) The wall thickness, rupture pressure, and suture strength of iPSC–SDBV are comparable to those of native HUAs (two‐tailed unpaired Student's *t* test was performed; dots indicate the values of results for individual hiPSC–SDBVs or HUAs). Immunofluorescence staining images of macrophage markers (CD68 and EC markers [CD31, vWF] 30 days after transplantation in vivo. iv) Nuclear was counterstained by DAPI. Scale bar, 100 μm. Reproduced with permission.^[^ ^98^ ^]^ Copyright 2020, Elsevier.

Qyang's group^[^ ^97^ ^]^ seamlessly combined iPSC‐derived SMCs with tissue engineering scaffolds to construct SDBVs for transplantation (**Figure** **8**A). These early hiPSC–SMCs showed expression of specific markers similar to those of primary human aortic SMCs (HASMSs) including α‐SMA, SM22α, SM–MHC, and calponin. α‐SMA and SMMHC localization after 14 days of abdominal aortic transplantation showed extensive vascular remodeling in vivo. However, it was difficult to resist the radial expansion of blood flow due to the low mechanical strength in the rat aortic model. Therefore, they did not meet the clinical transplantation conditions. The team then placed the PGA scaffold inoculated with iPSC–SMCs in a bioreactor with pulsatile flow (Figure 8B).^[^ ^98^ ^]^ Mechanically robust hiPSC–SDBVs were obtained by mechanical stretching to promote collagen deposition and SMC proliferation. After 8 weeks of mechanical exercise, the mechanical properties of iPSC–SDBVs were equivalent to those of natural vessels and still maintained the characteristics of lumen. Endothelial characteristic markers showed that the graft still displayed vascular remodeling biological activity.

#### 3.2.3 SMCs Derived from ESC

A safe and feasible strategy to obtain a large number of functional SMCs is to drive ESCs to differentiate into the SMCs in vitro. ESC‐derived SMCs are a promising cellular source for vascular tissue engineering.^[^ ^99^, ^100^ ^]^ ESCs should be cultured under specific culture conditions to differentiate into SMCs such as medium composition, growth factor addition, and lineage origin specificity. The spatial localization of SMCs in embryonic development is not confined to the mesoderm (lateral plate mesoderm, paraxial mesoderm, etc.), but may also originate from the neural crest of the neuroectoderm.^[^ ^101^, ^102^ ^]^ Na's group^[^ ^77^ ^]^ developed a simplified and chemically defined medium to accelerate the metabolism and mesoderm response of ESCs. Notably, that lineage‐specific SMCs also have a functional tendency. Studies have shown that side plate mesoderm‐derived SMCs upregulate ECM degradation while simultaneously being substantially enriched in genes that promote cell migration, resulting in maximum migration. In contrast, neuroectoderm‐derived SMCs contain cell groups associated with myelination and synaptic transmission.^[^ ^103^ ^]^

## 4 SDBV Manufacturing Based on 3D Bioprinting

The 3D bioprinting can precisely control the distribution of cells and materials in a 3D environment, which has obvious advantages in bionic three‐layer structures of blood vessel walls with different cell distributions.^[^ ^104^, ^105^, ^106^, ^107^ ^]^ Several studies related to 3D‐printing SDBVs are displayed in **Table** **2**.

**Table 2:** Constructing SDBVs with 3D printing.

Cell type	Construction method	Main functions	References
HUVECs, MSCs	Cell‐responsive bioink and multilayer coaxial extrusion systems were used in one step depending on the diffusion and proliferation of endothelial cells and stem cells encapsulated in a bioprinting structure to form a highly organized and perfusable vascular structure with biological activity.	Support the proliferation and early maturation of vascular cells and the occurrence of complex vascular structures.	Zhang et al.^[^ ^124^ ^]^
HUVECs, HUASMCs	Construction of an imitating venous catheter by co‐printing or inoculating cells with stretchable double‐network hydrogel bioink.	The arteriovenous vascular structure allows primary vascular anastomosis in vitro with the human popliteal vein or mouse aorta and in vivo vascular anastomosis in the mouse vena cava.	Zhang et al.^[^ ^125^ ^]^
EPCs	Bioactive blood vessels were designed by 3D coaxial cell printing technology and a bioink composed of VdECM and alginate loaded with EPCs was used.	The perfusable vessels loaded with EPCs in the hind limb ischemia model of nude mice improved the recovery rate of ischemic injury.	Cho et al.^[^ ^123^ ^]^
MSC–ECs	MSCs were differentiated into endothelial‐like cells and then inoculated onto a PCL scaffold constructed by 3D printing to construct artificial blood vessels with three layers.	Autologous MSCs induce endothelialization on the lumen surface of artificial blood vessels and it is difficult to cause acute thrombosis after transplantation in a dog model.	Youn et al.^[^ ^119^ ^]^
ECs, SMCs	SMCs and nano‐engineered bioink were co‐encapsulated and printed onto ECs. A multilayer vascular structure with a healthy phenotype was inoculated on the surface of the intracavity	As a potential tool for understanding the pathophysiology of vascular diseases, the 3D bioprinted blood vessel can summarize the events of thrombotic reactions for drug evaluation and treatment in preclinical trials.	Gaharwar et al.^[^ ^115^ ^]^
HUVECs, SMCs	One‐step preparation of a small‐diameter heterogeneous bilayer vascular‐like structure by loading SMCs and ECs with different concentrations of bioink combined with 3D micro‐extrusion bioprinting.	The heterogeneous double‐layer vascular‐like structure represents a new strategy for the biological manufacture of small‐diameter blood vessels.	Atala et al.^[^ ^20^ ^]^
ECs, SMCs	Creation of bionic and adjustable small‐diameter blood vessels with an endodermis and muscular‐layer using 3D coaxial printing	The interaction between material degradation and cell migration leads to a good premise of angiogenesis, which may be a potential substitute for the clinical application of small‐diameter vessels	Zhang et al.^[^ ^116^ ^]^
MSCs	The circumferential fiber scaffold was designed to biomimetic autologous vascular compliance and provide suitable microcues for MSC proliferation and migration to realize the early accumulation of ECM components in the scaffold	The design of ECM components in the bionic body of the fiber structure realizes the close combination of the structure–function relationship by reforming mechanical energy	Klein et al.^[^ ^152^ ^]^

### 4.1 3D Bioprinting Provides Spatial Cues for Cell Proliferation

Through precision deposition technology, 3D printing overcomes the shortcomings of traditional scaffold construction methods, such as poor control of the scaffold's microstructure.^[^ ^108^ ^]^ Ruan's group describes for the first time a bioink that utilizes electrostatic self‐assembly to release biomolecules sequentially in space–time coordination for coaxial 3D extrusion printing to construct polyelectrolyte SDBV (diameter > 1.3 mm).^[^ ^109^ ^]^ Polyelectrolyte bioink consists of negatively charged sodium alginate (SA) and positively charged ε‐polylysine, on which heparin and pentapeptide Tyr–Ile–Gly–Ser–Arg (YIGSR), functional motif of laminin) are adsorbed by electrostatic interaction. Heparin was first and released in the whole life cycle of SDBVs, thus preventing blood biomaterials from causing thrombosis. EC adhesion mediated by YIGSR was initiated with heparin delivery. Despite this, the functional maintenance of SDBV in actual blood perfusion remains to be investigated.

With increasing attention paid to the biological activity of SDBVs, the concept of vascular cell loaded has gradually been introduced into SDBV structures built by 3D printing. Song's group^[^ ^110^ ^]^ used gelatin, SA, and carbon nanotubes as bioinks to construct biomaterial scaffolds with 3D extrusion printing technology (diameter > 3 mm). Fibroblasts, SMCs, and ECs were then introduced into the lumen layer by layer. The introduction of cells was intended only to demonstrate the biocompatible properties of the scaffold and thus to evaluate the potential viability of the graft. Aiming at the cell‐friendly multilayer tubular structure manufacturing, Zhang's group proposed a method for multichannel coaxial extrusion system (MCCES) by digitally coded.^[^ ^111^ ^]^ SMCs and ECs could be cocultured by double‐layer vascular tissue, which enables the formation of a monolayer endothelium within 14 days. The advantage of MCCES lies in tunable tube diameter (diameter: ≈0.7 mm), customizable tissue layers and loading cell types, which provide an available way to prepare SDBVs with different vascular types.

Researchers are increasingly focused on directly suspending cells uniformly in bioinks and depositing them simultaneously with biomaterials in particular areas to construct spatial structures, as well as inoculating cells in 3D scaffolds.^[^ ^112^ ^]^ Ye's group studied the effect on the microstructure and activity of human dermal fibroblasts by varying the concentration and heat treatment duration in bioinks (diameter: 4.9 mm).^[^ ^113^ ^]^ The findings demonstrated that a longer heat treatment duration resulted in a loose porous microstructure in the low concentration of gelatin and a trend toward increased cell viability. Therefore, the regulation of 3D printing on the microstructure of bioink occurs by creating spatial sites suitable for cell proliferation, hence improving the biological activity of tissue‐engineered blood vessels. To further solve the problem of low activity caused by shear stress of loaded vascular cells in 3D printing, Ouyang's group put forward the concept of “in‐situ crosslinking” by improving the general 3D printing, that is, cross‐linking hydrogel instantly before deposition to achieve high cell activity and structural uniformity.^[^ ^114^ ^]^ The design takes advantage of the fact that rapid and cell‐compatible gelation occurs within the capillaries in the case of simultaneous extrusion and cross‐linking, thus possibly protecting cells from high shear stress. And in situ cross‐linking was universal for a series of photo‐cross‐linkable hydrogels. High‐resolution fabrication of cell‐loaded SDBV structure (diameter: 0.06–0.7 mm) was realized by using in situ cross‐linked coaxial extrusion 3D printing.

### 4.2 Precise Spatial Positioning for Multicell Types

**Figure 9:**
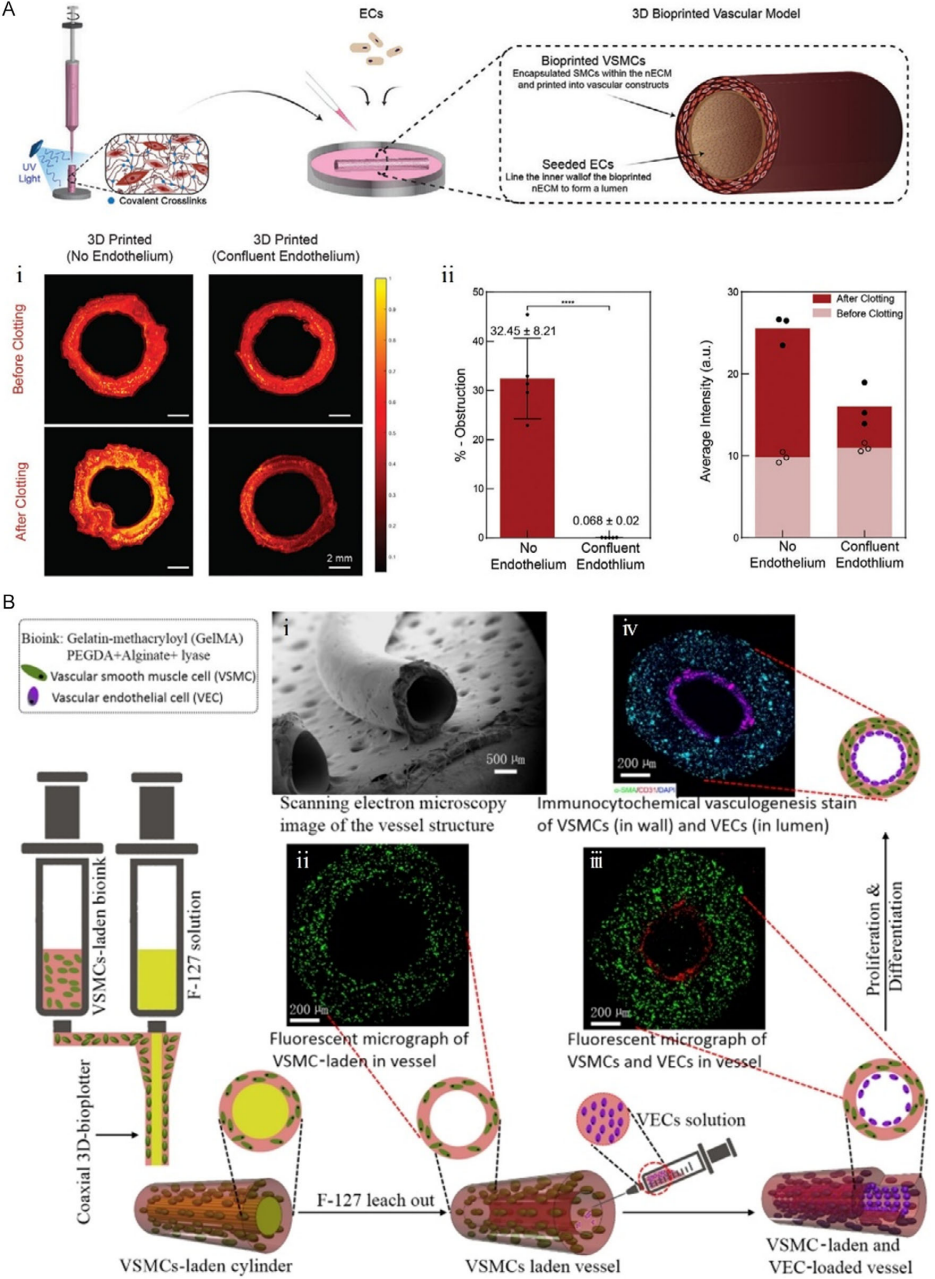
The construction Strategy of SDBV with multicell layer structure by 3D printing. A) Manufacturing strategy of bioprinting multilayer blood vessels. Coculture of VSMCs and ECs by 3D bioprinting blood vessel model. i,ii) Stenosis response of 3D‐bioprinted vascular model (no or confluent endothelium). Scale bar, 2 mm. Reproduced with permission.^[^ ^115^ ^]^ Copyright 2021, John Wiley and Sons. B) Schematic diagram of manufacturing small‐diameter blood vessels with bionic biphasic cell layer by 3D printing. i) Scanning electron microscopy image of the vessel structure. ii) Fluorescent micrograph of VSMC laden in vessel. iii) Fluorescent micrograph of VSMCs and VECs in vessel. iv) Immunocytochemical vasculogenesis stain of SMCs (in wall) and VECs (in lumen). Scale bars, (i) 500 μm and (ii–iv) 200 μm. Reproduced with permission.^[^ ^116^ ^]^ Copyright 2020, American Chemical Society.

Multiple cell types are able to exhibit a consistent spatial distribution on 3D‐printed vascular scaffolds. Gaharwar's group designed a nanoengineered ECM made of GelMA, poly (ethylene glycol) diacrylate (PEGDA), and 2D nano silicates that may be utilized to fabricate distinct vessels of different diameters immediately after encapsulating SMCs.^[^ ^115^ ^]^ When ECs are injected in the inner layer and confluent into a monolayer, cocultured vascular models with SMCs are formed (**Figure** **9**A). When blood was perfused into vessels without ECs, a more significant degree of coagulation was observed by OCT than in vessels with ECs. Furthermore, one limitation of this is the inability to confine ECs to certain inner surfaces, resulting in inefficient cell seeding, thereby leading to the failure of vascular grafts under sustained blood flow. Similarly, in 3D coaxial printing based on extrusion, Zhang's group^[^ ^116^ ^]^ used a biodegradable GelMA, PEGDA, and alginate mixed hydrogel as a bioink, loaded vascular smooth muscle cells (VSMCs) to form the blood vessel wall (diameter: 1.2 mm), and then achieved the construction of hollow pipes through the dissolution of Pluronic F‐127, which supports the lumen during the printing process (Figure 9B). Vascular endothelial cells (VECs) enter the inner side of the lumen to create a new bionic blood vessel with two distinct cell layers. Yet, the dissolution of F127 used in this strategy can lead to the change of osmotic pressure of biological ink, which would interfere with the structural accuracy and cell viability of SDBV.

On this basis, Zhao's group proposed a two‐step cross‐linking method to construct SDBV (diameter: 0.08–0.2 mm).^[^ ^117^ ^]^ The one‐fourth tube wall was 3D‐printed and cured by short‐term ultraviolet irradiation, which combined with concave uncross‐linked GelMA hydrogel to form a double‐layer SDBV structure. Analogously, SMC was loaded with bioink, and EC was implanted on the inner wall of the SDBV by perfusion. This design avoids the short board of sacrificing materials. Unfortunately, the improvement of SDBV perfusion performance and biological activity by SMC‐ and EC‐loaded bilayer structure were not involved in the study. However, most of the EC suspension passed through the blood vessels and was dispersed in the medium, with only a few colonizing the lumen.

Hence, compared to the method of adding other cell types via additional steps after printing, 3D‐printed bioink loaded with cells achieves integrated functioning and ensures precision of cell spatial positioning.^[^ ^20^ ^]^ Roman's group^[^ ^118^ ^]^ constructed a double‐layer vascular‐like structure (diameter: 1.5 mm) using triple coaxial printing technology, and HUVECs and HASMCs were individually encapsulated to keep them independent in each layer. This is the first attempt to encapsulate both types of blood vessel cells in separate layers while simultaneously extruding various tubular structures.

## 5 3D‐Printed Stem Cells for the Construction and Maturation of SDBVs

The use of 3D‐printed scaffolds to provide spatial cues for the multilineage differentiation of stem cells has increased with the field of SDBVs. This has effectively limited the acquisition of multiple cell types and vascular stents to the same time.

Youn's team constructed a 3D‐printed SDBVs model (diameter: 4 mm) loaded with MSC‐derived ECs for canine model validation.^[^ ^119^ ^]^ The loaded cell group had significantly higher endothelial coverage than the control group (61.9 cells mm^−2^ ± 14.3 [cell‐derived group] versus 21 cells mm^−2^ ± 11.3 [control group]). However, in the canine model, the patency rate of blood vessels containing autologous cells was only marginally greater than that of the control group. The reduced patency rate could be attributed to poor maintenance of MSC–EC function in the bioink or culture environment. Ulteriorly, Zhang's group introduced SMCs and ECs derived from iPSC into SDBVs (diameter: 4 mm) for co‐printing.^[^ ^120^ ^]^ GEL–LAY PORO–LAY (Gel‐Lay) elastomer provides strong mechanical properties for SDBVs while fibrin gel with more biological activity is used as a matrix for SMCs and ECs in the middle layer and inner layer, respectively. The materials and cell components were combined to form SDBVs by frequency‐division multiplexing 3D printing. Both iPSC‐derived SMCs and ECs showed obvious growth and expansion to form a double‐layer structure with active myogenesis and angiogenesis after 7 days of 3D printing. In summary, this study highlights the well‐known advantages of stem cell‐derived vascular cells in proliferation ability and anti‐senescence in culture.

Studies have revealed the possibility of directly incorporating stem cells or progenitor cells into bioinks for 3D printing. Endothelial progenitor cells (EPCs) are a broad name for numerous cell types that could differentiate into endothelial lineages, SMCs, and pericytes.^[^ ^121^ ^]^ The pre‐inoculation of EPCs to build an antithrombotic barrier has become a means for increasing vascular graft patency.^[^ ^122^ ^]^ Cho's group combined 3D coaxial printing with vascular‐tissue‐derived decellularized ECM (VdECM) and alginate‐mixed bioink‐loaded EPCs, to construct a functional SDBVs (diameter: 1 mm) for the treatment of ischemic diseases.^[^ ^123^ ^]^ As a result, EPCs localized on the tube wall were observed to spontaneously organize and form a monolayer endothelium with a continuous structure. The highly active endothelializing properties of EPCs confer the potential of vascular grafts to repair ischemic injuries.

**Figure 10:**
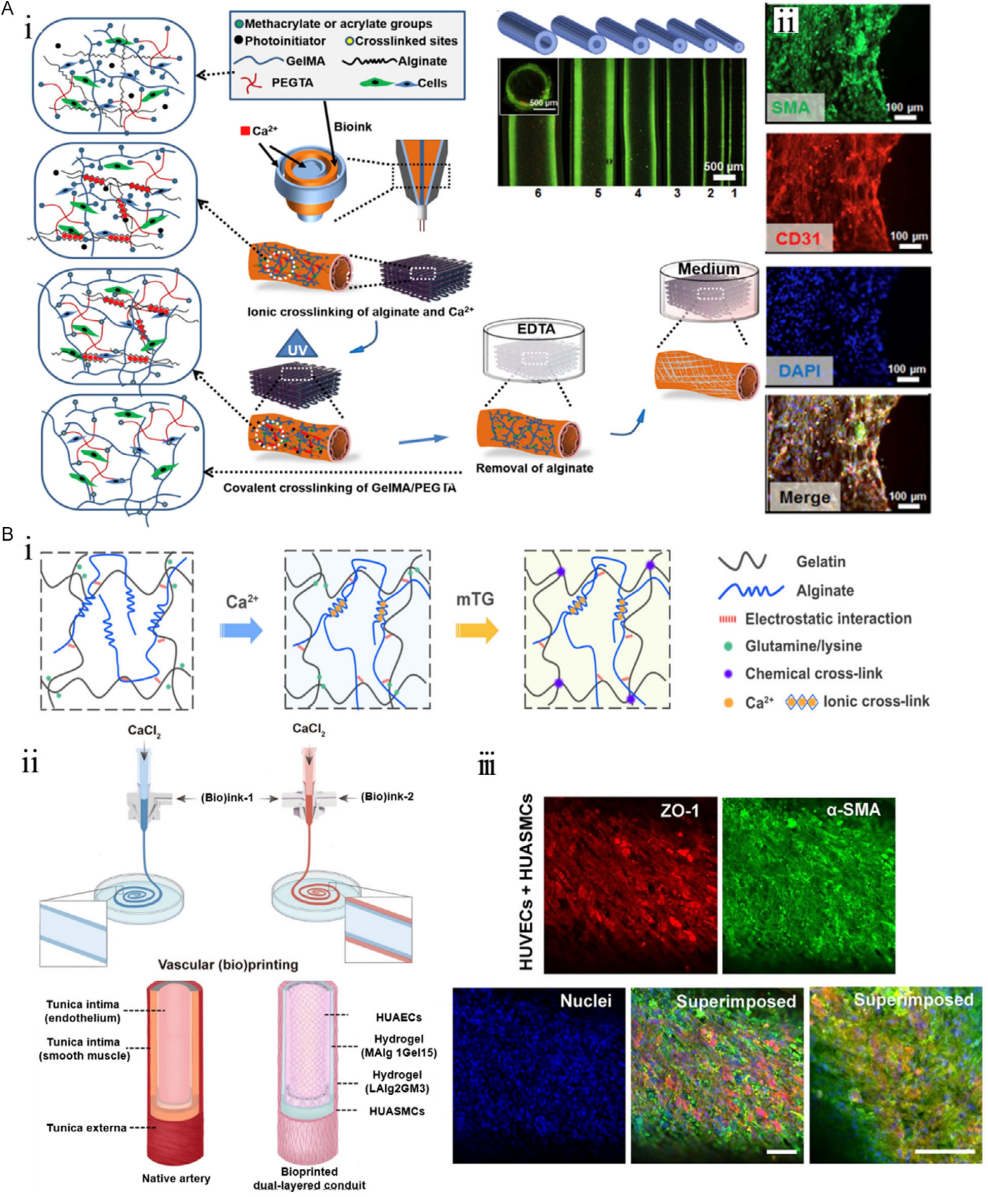
3D‐printed stem cells for construction and maturation of SDBVs. A) i) Multilayer coaxial extrusion printing is combined with dual‐stage cross‐linked bioink with ionic cross‐linking and covalent cross‐linking to form blood vessel structure. ii) The confocal micrographs of α‐SMA‐expressing MSCs and CD31‐expressing HUVECs 21 after printing. Scale bar, 100 μm. Reproduced with permission.^[^ ^124^ ^]^ Copyright 2016, Elsevier. B) i) Schematic diagram of construction of high‐strength dual‐network hydrogel by ionic cross‐linking and enzyme cross‐linking. ii) Schematic diagram of reconstruction of artery‐like structure by microfluidic bioprinting technology. iii) Fluorescence confocal images of the bioprinted artery show that zona occludens 1 (ZO‐1, red) localized to HUAEC and α‐SMA (green) localized to HUASMC. The nucleus (blue) was re‐stained with DAPI. Scale bars, 100 μm. Reproduced with permission.^[^ ^125^ ^]^ Copyright 2022, American Association for the Advancement of Science.

Zhang's team has successfully constructed a series of functional SDBVs using 3D‐printing technology. Previously, multilayer coaxial extrusion printing was combined with bioink, which coexisted with ion cross‐linking and covalent cross‐linking to create vascular structure (diameter: 1–6 mm)^[^ ^124^ ^]^ (**Figure** **10**A). After 21 days of culture, F‐actin localization showed that the tube wall with two‐stage cross‐linking components had great potential to form a vascularized complex tissue structure. The results of CD31 and α‐SMA immunofluorescence staining revealed the effect of 3D‐printed blood vessels on maintaining the activity of HUVECs and promoting the differentiation of MSCs into functional cells. The dual‐stage cross‐linking mechanism of bioink has also been studied.^[^ ^125^ ^]^ A high‐strength dual‐network hydrogel bioink was constructed using ion cross‐linking and enzyme cross‐linking (Figure 10B), and artery‐like structures (diameter: 1 or 5 mm) were reconstructed using microfluidic bioprinting. The inner and outer hydrogel enclosing the human umbilical vein SM cells (HUVSMCs) were bioprinted, followed by a culture cycle of at least 14 days until the HUVSMCs were relatively mature, and the HUAEC was further inoculated into the tube by perfusion again. After another 7 days of culture, an arterial‐like structure with an endodermis was obtained. The correct localization of zonula occludens 1 and α‐SMA in HUAECs and HUASMCs, respectively, reveals the formation of arterial lumens with mature endothelia and muscular layers. The results of the in vitro perfusion of the mouse aorta and human popliteal vein showed no leakage for a short duration, which was consistent with the results of the in vivo anastomosis of the mouse vena cava at the later stage. However, for vascular cells derived from stem cells encapsulated in 3D printing, the long culture cycle remains an urgent research challenge that needs to be broken through.

Furthermore, the combination of 3D printing and bioinks that encapsulates ESCs or iPSCs with notable differentiation potential holds a rosy prospect in the field of SDBVs. Generally speaking, the combined application of 3D printing and stem cells is still preliminary in the field of SDBV construction. Gratifyingly, it has been used in various fields of tissue engineering, including but not limited to lung regeneration,^[^ ^126^ ^]^ spinal cord repair,^[^ ^127^, ^128^ ^]^ skin healing,^[^ ^129^, ^130^ ^]^ bone repair,^[^ ^131^, ^132^, ^133^, ^134^, ^135^, ^136^ ^]^ periodontal tissue construction,^[^ ^137^ ^]^ endometrium repair,^[^ ^138^ ^]^ cardiac repair,^[^ ^134^, ^139^ ^]^ peripheral nerve repair,^[^ ^140^ ^]^ and vascularized liver tissue construction, among others, verifying the reliability and broad prospects of the combination.

## 6 Future Perspectives

In most clinical cases, a lack of excellent and extensive sources of autologous grafts persists, and the use of allografts is limited due to immunogenic rejection of recipients.

To resolve this contradiction, the combination of various engineering strategies and stem cells is an excellent practical way to manufacture the small‐diameter vascular grafts which meet the basic requirements.

Compared with the scaffold of vascular tissue engineering, the SDBVs from 3D printing are closer to the layered structure of the autologous branch, to achieve a situation of bionic structure. They are also more suitable for the clinical requirements for manufacturing efficiency in the integrated construction of the three‐layer structure of blood vessels. However, SDBVs built by 3D printing still have some problems to solve in the future. Mechanical strength is difficult stabilize in vivo; therefore, vascular stents should still be mechanically stable under the scouring of blood flow according to the needs of different graft sites, especially the anastomosis site between the graft and autologous blood vessel. A lack of mechanical strength often leads to the stenosis, dilation, or rupture of local blood vessels, which leads to the failure of transplantation.

To further enhance the availability of SDBVs, incorporating host ECs and SMCs could be beneficial to achieve a win–win situation between mechanical enhancement and vascular biological activity. Among the cell sources used for tissue engineering blood vessels, stem cells have become the most potential primary cell substitutes for research and application. Notably, MSCs, iPSCs, ESCs, and other progenitor cells can differentiate into specific vascular cell types in vitro. Compared with primary vascular cells, these stem‐cell‐derived vascular cells may have stronger proliferative characteristics and an equivalent contractile phenotype under specific culture conditions. Therefore, stem cells with rapid proliferation and multiline differentiation ability play an important role in timely treatment.

The composite application of 3D printing and stem cells complements each other. On the one hand, the microstructure present in 3D‐printed vascular scaffolds provides special sites for the proliferation and differentiation of stem cells to realize the transformation to a specific cell fate. On the other hand, stem cells play the role of specific cell types through maturation on 3D‐printed vascular scaffolds, thus simultaneously promoting the biological activity and mechanical enhancement of SDBVs. For example, the maturation of SMCs derived from stem cells represents the deposition of more ECM to provide additional mechanical support for SDBVs; ECs derived from stem cells are an important bioactive component for maintaining patency and resisting thrombosis.

However, there are still some crucial problems that have become limited links in the 3D printing of SDBVs derived from stem cells. Most current research focuses on the construction of SDBVs by co‐printing or inoculating differentiated vascular cells derived from stem cells after printing. To this date, no study has addressed the direct printing of stem cells and bioink to realize integrated construction. A possible reason for this is that the differentiation scheme of stem cells into vascular cells is extensive and open at present, and the use of various small molecules or culture medium components are characterized by lack of universality, which makes it difficult to guide the differentiation of vascular cells in a standardized and effective way. In addition, to realize the direct 3D printing of loaded stem cells, it is with urgency that the manufacture of bioink should be specialized. Since stem cells are highly susceptible to spontaneous differentiation, bioinks are required to maintain stemness. In addition, research into bioink should be based on the recent influence of 3D micro on stem cell differentiation.

In short, 3D‐printed stem cell‐derived SDBVs simplify the preparation and stability of SDBVs, which will have wide applications in regenerative medicine and tissue engineering. Although certain limitations still persists, the combined application of 3D printing and stem cells still has unlimited potential. In the future, SDBVs based on both of these will contribute to human vascular tissue engineering from experimental research to clinical application.

## Conflict of Interest

The authors declare no conflict of interest.

## Author Contributions

**Yifan Wang**: Investigation (equal); Writing—original draft (equal). **Xinhuan Wang**: Investigation (equal); Writing—original draft (equal). **Jun Chen**: Conceptualization (lead); Funding acquisition (lead); Supervision (lead); Writing—original draft (lead); Writing—review & editing (lead). **Gordon Wallace**: Conceptualization (equal); Writing—review & editing (supporting). **Qi Gu**: Conceptualization (lead); Investigation (lead); Writing—original draft (supporting); Writing—review & editing (lead). **Yifan Wang** and **Xinhuan Wang** contributed equally to this work.
